# Design Implications of Comfort and Usability of Manual Stairclimbing Wheelchair: Ergonomic Assessment and Pilot Study Using Surface Electromyography Inputs

**DOI:** 10.2196/78965

**Published:** 2026-04-13

**Authors:** Abhishek Verma, Rohit Kumar, J Ramkumar

**Affiliations:** 1 Department of Design, Indian Institute of Technology Kanpur, DJAC Building, Kanpur, 208016, India, 91 9451220918

**Keywords:** stairclimbing wheelchair, ergonomic design, surface electromyography, taguchi design, maximum voluntary contraction, likert scale

## Abstract

**Background:**

Stairclimbing wheelchairs offer enhanced mobility for users navigating multilevel environments, yet limited research addresses the ergonomics of lever propulsion-based stair climbing mechanisms. Comprehensive ergonomic assessment integrating both subjective user feedback and objective biomechanical analysis is essential for optimizing assistive device design for comfort and usability.

**Objective:**

This pilot study aims to assess the ergonomic design of a transformable stair-climbing wheelchair through a dual-methodology approach, evaluating plane surface movement accessibility and quantifying muscle activation patterns during lever-propelled stair-climbing operations using surface electromyography (sEMG).

**Methods:**

This 2-part study involved anthropometric measurements from 20 male participants to establish design parameters using 5th and 95th percentile values. Part A assessed plane surface movement with 9 participants (7 healthy, 2 with paraplegia) navigating a simulated urban course featuring a 5° ramp, a 90° turn, and narrow passages across 3 trials. Task completion times and subjective ride easiness ratings were recorded. Part B used a Taguchi-based fractional factorial design to evaluate 3 ergonomic factors, including torso angle (λ), lever distance (L), and lever orientation (ψ), across 7 healthy participants. Maximum voluntary contraction (MVC) was measured for 4 muscles, including biceps brachii long head (BBL), triceps brachii long head (TBL), brachioradialis, and posterior deltoid (PDT).

**Results:**

In Part A, the ramp and 90° turn proved most challenging due to the wheelchair’s 65 kg weight and large turning radius (~1450 mm). Driving control scored highest (6/10), while comfort scored lowest due to the tilted seat design. In Part B, a straight torso (λ=0°) consistently reduced muscle strain, particularly for brachioradialis. A lever distance of approximately 50 mm and a neutral to slightly supinated orientation (ψ=0°-30°) optimized muscle effort. Interaction effects revealed high strain configurations (λ=45°; L=100 mm; ψ=−30°) exceeding 75 MVC, while optimal settings reduced strain to approximately 50 MVC.

**Conclusions:**

Optimal ergonomic parameters of λ=0°, L=37.5 mm, and ψ=15° are recommended to minimize fatigue and enhance user comfort. Design improvements should prioritize weight reduction, compact form factor for maneuverability, and adjustable seat tilt. The modular wheelchair design permits customization for diverse user populations. Future research should include larger, gender-diverse participant groups and real-world validation studies.

## Introduction

Ergonomic analysis of any newly designed wheelchair or assistive technology intervention is crucial for assessing user comfort and efficiency using already established methodologies. Subjective evaluations, such as Likert scales and visual analog scale (VAS), are used to evaluate user perception about usability and comfort of an assistive technology. Cowan et al [1], assessed paraplegic users, using questionnaires on maneuverability, stability, and comfort, alongside time and kinetic data, providing a comprehensive view of user experience. Van der Woude et al [2], reviewed research and innovation in manual wheelchairs, focusing on rehabilitation, sports, daily life, and health, emphasizing physiological responses during propulsion. However, most studies focus on flat surfaces, with limited research specifically addressing the ergonomics of lever-propulsion–based stair-climbing, indicating a gap that this study aims to address. Dubowsky et al [3] compared kinematics, kinetics, and electromyography in able-bodied and paraplegic users, analyzing biceps brachii and triceps brachii during propulsion, highlighting axle placement effects.

Studies on specific design variables that may affect muscle activity are crucial for design optimization. Marrow et al [4] examined the influence of grip position on upper-extremity posture and muscle activity, linking pronation, supination, and neutral positions along with torso inclination to muscle demands. Kurup et al [5] looked at push handle height, affecting the distance from the shoulder to the lever, and its impact on muscle activity. Verma et al [6] reviewed how different ergonomic and functional considerations inform manual wheelchair design, emphasizing wheelchair dimensions based on the stair-climbing mechanisms. Among all these studies based on the wheelchair functional or comfort assessments, there is an implicit absence of pilot studies on newly developed stair-climbing technologies that use electromyography to enhance comfort and usability, highlighting the role of electromyography in designing for reduced muscle strain. Such a type of approach has been carried out in this research to discuss implications of integrating electromyography data into design processes for minimizing muscle strain and improving overall user experience in the case of stair-climbing operations.

The ergonomic evaluation of stair-climbing wheelchair functions is necessary for further iterations into the wheelchair design developed by Verma et al [7]. For an assistive technology such as this stair-climbing wheelchair, the goal is to offer assistance in activities of daily living such as mobility, nutrition, personal care, and employment and also act as an enabler of social interactions such as family or relationship responsibilities and interpersonal or community relationships, etc. Both subjective and objective information about the effectiveness of the wheelchair is required for improvement in this design of the stair-climbing wheelchair for both plane surface movement and stair-climbing function. This research focuses on understanding the wheelchair use case for a short duration. Any long-term consequences of the wheelchair design require a more detailed study spanning over years and are out of the scope of this research. This research can be broadly divided into 2 parts, the first one to collect and assess wheelchair mobility on a plane surface when the wheelchair is transformed into a plane-surface wheelchair, and the second part addresses the ergonomics of stair-climbing when the participant performs the pull-push action through a set of levers to rotate stair-climbing wheel.

This pilot study uses an integrated dual-methodology approach to comprehensively evaluate the stair-climbing wheelchair design. Part A focuses on ergonomic assessment of plane surface movement through anthropometric measurements, functional mobility testing, and subjective user feedback. Part B complements this assessment through objective biomechanical analysis using surface electromyography (sEMG) to quantify muscle activation patterns during stair-climbing operations. These 2 methodological components are mutually reinforcing; the subjective assessments from Part A identify user-perceived comfort and usability issues, while the objective sEMG measurements from Part B provide physiological validation of these subjective experiences and reveal underlying biomechanical factors that may not be immediately apparent to users. This integrated approach enables both immediate user feedback and deeper understanding of the physiological demands imposed by specific design parameters, facilitating evidence-based design optimization that addresses both perceived comfort and actual physical strain. The combination of these methodologies provides a comprehensive framework for assistive technology assessment that can inform future design iterations and clinical recommendations.

## Methods

### Study Design Overview

This 2-part pilot study used a dual-methodology approach to evaluate the ergonomic design of a transformable stair-climbing wheelchair. Part A assessed plane surface movement accessibility through anthropometric measurements, functional mobility testing on a simulated urban course, and subjective user feedback. Part B evaluated lever propulsion ergonomics during stair-climbing using sEMG to quantify muscle activation patterns under varying ergonomic configurations determined by a Taguchi-based fractional factorial design of experiments.

### Part A: Ergonomic Assessment of Plane Surface Movement

#### Participants and Anthropometric Measurements

**Figure 1.**
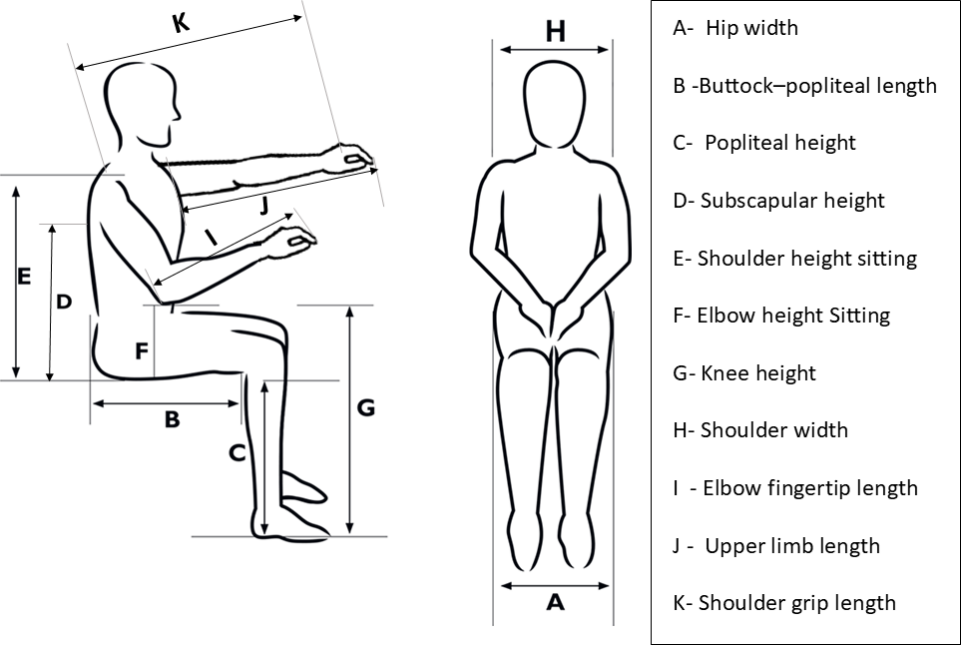
Anthropometric measurements are required to design wheelchair seating and reach dimensions.

Anthropometric measurements were collected from 20 male participants (average age 27.5, SD 2.76 years) to establish design parameters for the modular wheelchair. This demographic was selected to determine conservative upper-bound dimensions, recognizing that male participants generally have larger anthropometric dimensions. Measurements were taken with the user seated in an optimal position as shown in Figure 1. The dimensions collected included hip width, buttock-popliteal length, popliteal height, subscapular height, shoulder height, elbow height, knee height, shoulder width, elbow fingertip length, upper limb length, and shoulder grip length. These measurements were used to develop a fit and reach matrix according to user comfort, with 5th and 95th percentile values calculated for design purposes (Table 1).

**Table 1.** Anthropometric data recorded for wheelchair design (dimensions in centimeters).

Annotation	Anthropometric feature	Mean (SD)		5th -95th percentile	
—^a^	Age (years)	27.5 (2.76)		23.95-31.1	
—	Height (cm)	173.25 (3.46)		168-178	
—	Weight (kg)	71.75 (4.98)		63.95-78.15	
A	Hip width (cm)	38.45 (3.26)		34-42.15	
B	Buttock-Popliteal length	51 (4.41)		42.95-56.05	
C	Popliteal height (cm)	42.1 (2.67)		38.95-47.05	
D	Subscapular height (cm)	45.75 (2.22)		43-50.05	
E	Shoulder height (cm)	60.85 (3.28)		54.95-60.85	
F	Elbow height (cm)	24.85 (2.13)		22-28.05	
G	Knee height (cm)	53.95 (4.50)		48.95-60.15	
H	Shoulder width (cm)	55.45 (4.5)		50.85-63.15	
I	Elbow fingertip length (cm)	36.35 (2.75)		33-41.05	
J	Upper limb length (cm)	85.4 (4.28)		78.95-92.1	
K	Shoulder grip length (cm)	75.8 (5.01)		67.95-82.15	

a Not applicable.

#### Functional Anthropometry for Reach and Clearances

The wheelchair design dimensions were based on standards and guidelines from the Central Public Works Department (CPWD), the Office of Chief Commissioner for Persons with Disabilities, IS 4963 (building code standard), and IS 7454 (wheelchair specification standard). The clearance dimensions are summarized in Table 2.

**Table 2.** Clearance dimensions for the wheelchair based on standards and guidelines (in mm).

Reach ranges	Standards and guidelines			
	IS-7454^a^ [8]	CPWD^b^ [9]	CCD^c^ [10]	ISO^d^ [11]
Forward high	1350‐1600	1200	1200	1200
Forward low	Not specified	400	400	400
Forward obstructed high	715‐830	1100	1100	Not specified
Lateral high	1350‐1770	1300	1300	Not specified
Lateral low	Not specified	250	250	Not specified
Lateral obstructed high	Not specified	1200	1200	Not specified
Maneuvering clearances				
Circular diameter-360^o^ turn	1500	1500‐2000	1800	1500

a IS: Indian Standard in architecture.

b CPWD: Central Public Works Department.

c CCD: Chief Commissioner-Disability.

d ISO: International Standard Organization.

**Figure 2.**
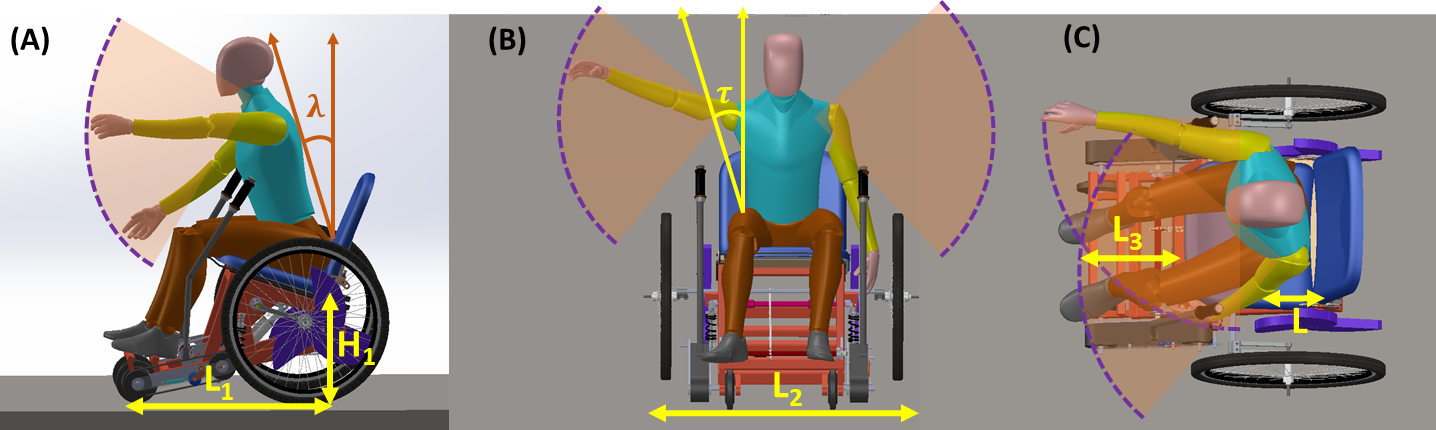
Maximum reach illustrated by a digital human model in (A) sagittal plane, (B) coronal plane, and (C) transverse plane.

The wheelchair dimensions for clearances and reach were based on the maximum of maximum (95th percentile) and minimum of minimum (5th percentile) human anthropometric dimensions, respectively. Wheelchair dimensions such as seat length and backrest angle were designed to be adjustable over the 5th and 95th percentile range using an angle-adjustable backrest and a slide-lock mechanism. The reach space design was based on wheelchair height (H1) for vertical reaches and length L1 as shown in Figure 2A-2B, width L2 (Figure 2B), and distance L3 (Figure 2C) for horizontal distances, calculated using a digital human model (DHM).

The primary reach relationships derived from the DHM are given by equations (1–4):

Forward high: (1)


$$H_{1}+E\times cos\lambda +K\times sin(\pi /4)\geq 1200mm$$

Forward low: (2)


H1+E×cosλ–K×sin(π/4)≥400mm

Lateral high:    (3)


H1+E+K×sin(π/4)≥1300mm

Lateral low: (4)


H1+E×sin(τ)–K×sin(π/4)≥250mm

Where H1 is the height of the wheelchair from the ground to the lowest point on the seat (500 mm), E is the shoulder height from the seat, *λ* is the torso angle the participants make with the coronal plane, K is the shoulder grip length, and *τ* is the angle the torso makes with the sagittal plane.

The minimum turning distance required for a 360° turn was calculated using Equation (5):

$T_{circular}=2\times \pi \times \left(\frac{L_{1}}{2}+\frac{L_{2}}{2}\right)$     (5)

Where $\left(\frac{L_{1}}{2}+\frac{L_{2}}{2}\right)$ is the turning radius of the wheelchair

For this prototype, L1=600 mm and L2=950 mm

Therefore, the distance required for a 360^o^ turn calculated using DHM will be,

T*_circular_*=4870 mm.

#### Simulated Course Design and Protocol

**Figure 3.**
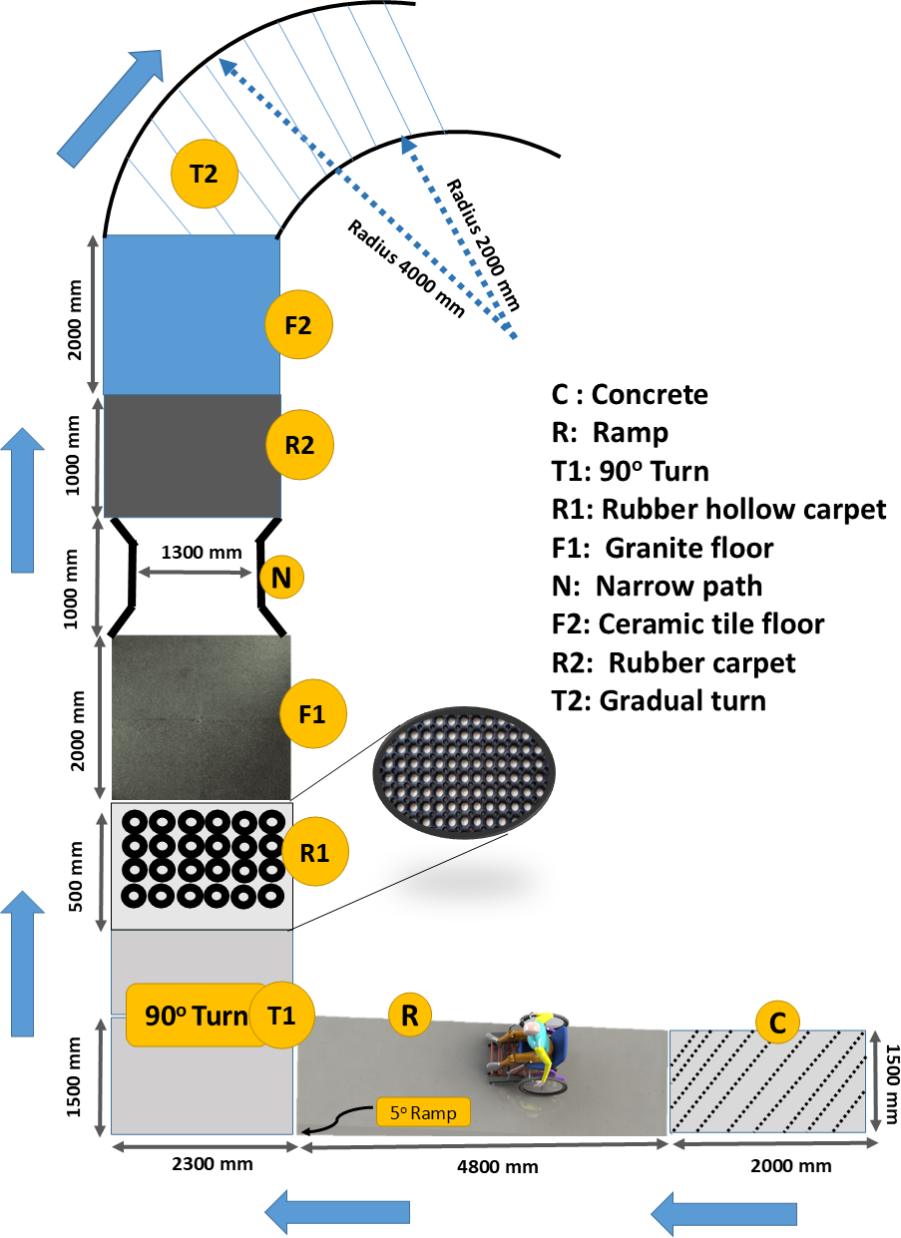
Wheelchair course designed for ergonomic design study of plane surface movement configuration of wheelchair.

The manual stair-climbing wheelchair is a dual-purpose wheelchair designed to provide multidimensional accessibility on both stairs and plane surfaces. To test the efficacy of wheelchair driving on plane surfaces, a simulated course was designed to test maneuverability and control. The simulated course contained architectural hurdles that a wheelchair user faces in an urban setting, including a 5° ramp followed by a sharp 90° turn and then converging and diverging spaces. The course is illustrated in Figure 3.

This pilot study used n=9 participants for plane surface assessment (7 healthy, 2 with paraplegia), following established guidelines for feasibility studies in assistive technology research [12,13]. Two participants with paraplegia provided critical real-world user validation. The course was designed as a controlled experimental environment to ensure participant safety and enable systematic data collection, including multiple stair configurations with varying heights (15‐20 cm) and depths (25‐30 cm), curved pathways, simulated obstacles, and various surface transitions representative of typical urban environments.

The subjective descriptors used for the response for each riding task using Likert scales were (1) strongly favorable (score=4), (2) moderately favorable (score =3), (3) moderately unfavorable (score=2), and (4) strongly unfavorable (score=1). Task completion times were recorded for each of the 9 tasks across 3 trials. After the completion of all tasks, a subjective survey of the whole task was taken on a visual satisfaction scale ranging from 0 (Very negative) to 10 (Very positive) for overall ride comfort, stability, driving control, perception, and maneuverability.

### Part B: Lever Propulsion sEMG Study

#### Muscles Under Study

The biomechanical evaluation of arm and shoulder muscles was performed to identify and mitigate shoulder pain and fatigue generated during lever propulsion. Use of sEMG is a prominent method for recording muscle activation artifacts to understand muscle effort, muscle activation timing, and muscle fatigue. The activation timing study of a muscle group was performed to understand the loading profile of muscle at a specific interval in the work cycle [14]. Surface electromyography also helps in identifying muscles under fatigue by observing an increase in amplitude and decrease in median frequency of the bioelectrical signal [15].

The upper limb and shoulder are the most common sites for any possible stress injury or musculoskeletal disorder due to long-term cyclic loading during lever-based propulsion [16,17]. The muscle groups under study for this specific research go under cyclic dynamic interactions and muscle fiber contractions during the lever-based propulsion cycle and are anisometric (concentric and eccentric) in nature. Therefore, the shoulder and arm joint angles are also required to be recorded while recording sEMG activity. The recorded electromyography signal has artifacts from the velocity- and angle-dependent muscle excitation of the shoulder and elbow extensors and flexors. The muscle activation of the extensor and flexor groups will be significant during both actions of pulling and pushing. But, since only pulling stroke is responsible for stair climbing, only those muscles will show significant dynamic electromyography activity that are associated with pulling action (elbow flexors and shoulder extensors muscle groups).

The action of lever propulsion in wheelchairs and its physiological effects are reported by only a few researchers, as the wheelchairs developed with a lever mechanism are very much limited, and in consequence, electromyography-based ergonomic evaluations based on them. There are few studies about lever-based propulsion effort, but they are limited to physiological measurements such as oxygen uptake, respiration ratio, minute ventilation, heart rate, etc [18-20]. With the advent of surface electromyography recording and processing techniques, the idea of using electromyography as a dominant physiological parameter that can influence design is increasing. Push-rim–based propulsion of a plane-surface wheelchair has been highly explored using surface electromyography studies on dominant-side muscle groups such as the deltoid group, triceps group, and bicep groups. As reported in the literature, there are some overlapping muscle groups engaged during both lever propulsion and hand-rim propulsion, such as biceps brachii, posterior deltoid, triceps, pectoralis major, etc [21-23]

The action of lever propulsion is a push-pull action that requires elbow (flexion and extension) and shoulder (flexion and extension) movement. The action of pulling requires flexion of the elbow and extension of the shoulder muscles and vice versa for pushing. The pulling stroke is responsible for rotating the stair-climbing wheel on the staircase. The pushing stroke (moving the lever away from the chest) is idle and requires less effort compared to pulling. Tomiak et al [24], in their research related to bimanual rowing movement, which mimics a lever push-pull activity, group together the pulling and pushing (returning) muscle groups as elbow and shoulder muscles. The primary goal of the ergonomic evaluation in this section is to identify extreme joint loading forces by trying different posture configurations defined by ergonomic factors. Pushing and pulling tasks can cause extreme lower back and upper joint loading, leading to musculoskeletal disorders (MSDs) [25,26].

**Figure 4.**
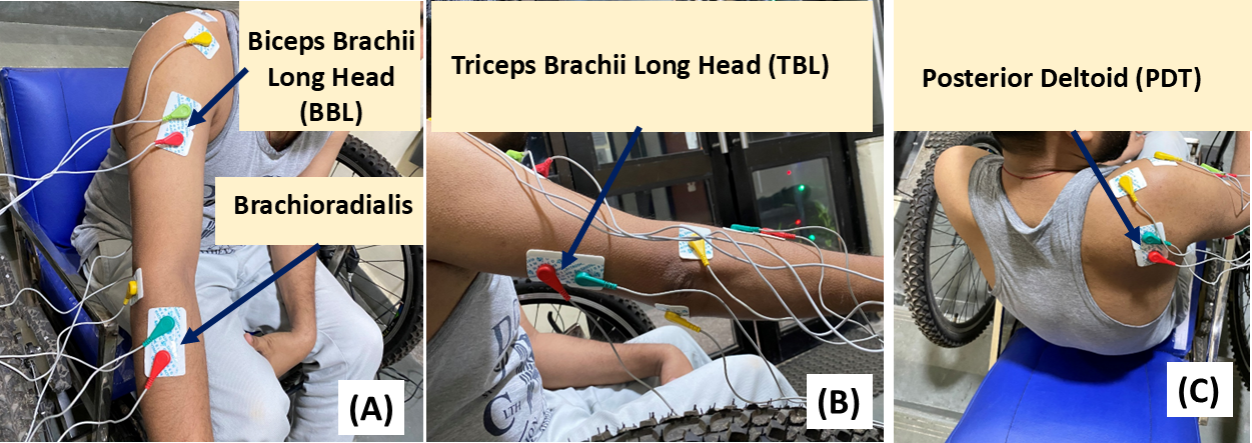
Nickel-chromium (Ni-Cr) alloy electrodes stuck on muscle surface using silver-silver chloride electrode (Ag-Cl) hydrogel (A) location of brachioradialis and BBL muscle group recording, (B) triceps brachii long head (TBL), and (C) posterior deltoid (PDT) muscle group recording.

Four muscle groups were selected for sEMG recording: (1) biceps brachii long head (BBL), a primary muscle involved in flexing the elbow, selected due to availability of higher density of motor neurons (Figure 4A); (2) triceps brachii long head (TBL), located on the back of the upper arm, responsible for extending the elbow during the pushing stroke (Figure 4B); (3) brachioradialis, responsible for forearm and elbow flexion necessary for pulling, with secondary function of supination. and pronation for holding the lever (Figure 4B); and (4) posterior deltoid (PDT), located at the shoulder, extends during shoulder extension required for pulling the lever toward the chest (Figure 4C).

#### Ergonomic Factors

**Figure 5.**
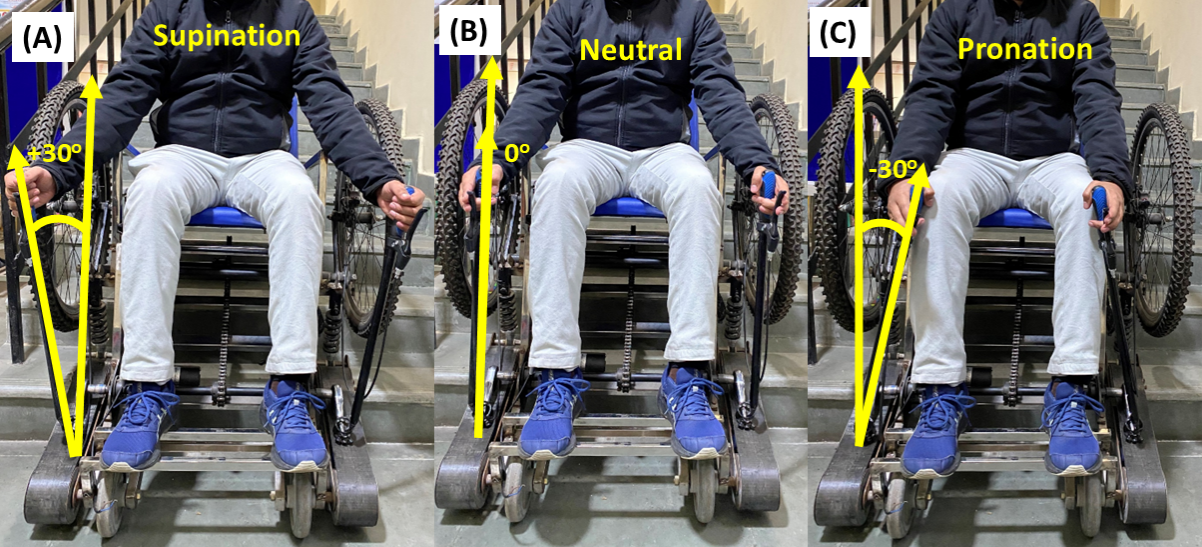
Lever orientation (ψ) as an ergonomic factor to study the effect of (A) supination, (B) neutral, and (C) pronation on muscle activation.

**Figure 6.**
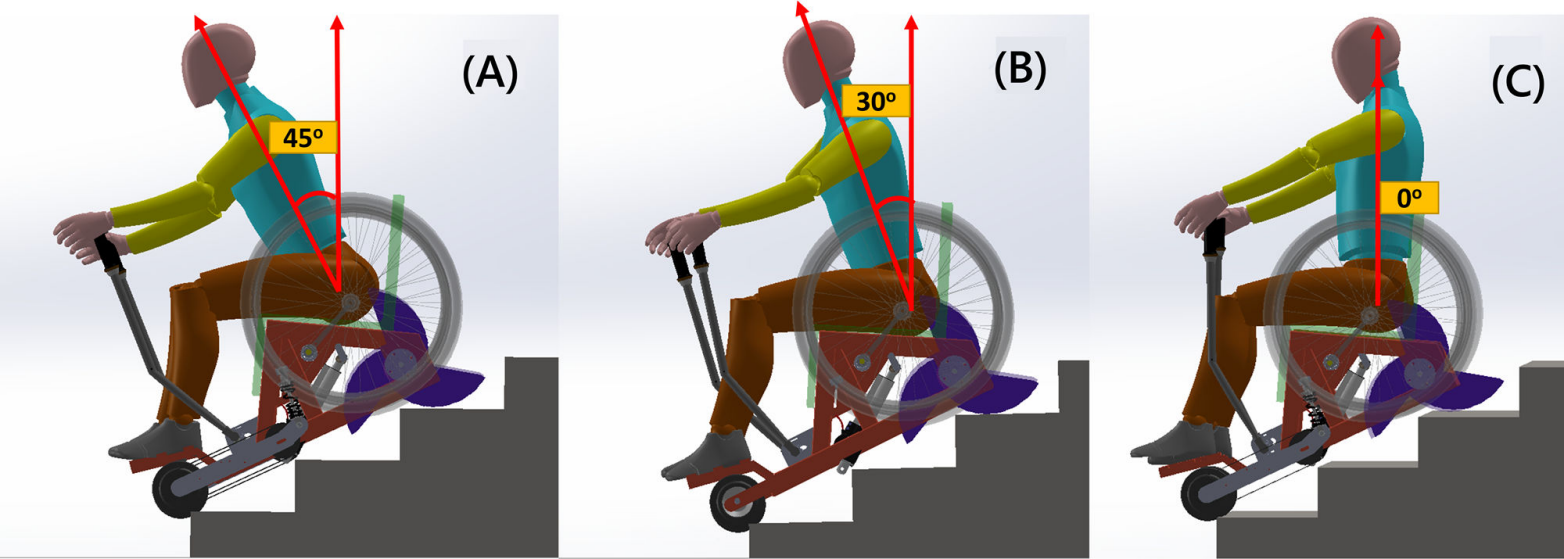
Torso angle made with vertical coronal plane (A) 45°, (B) 30°, and (C) 0°.

**Figure 7.**
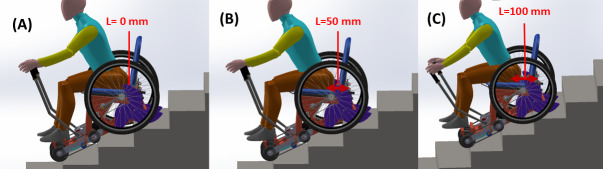
Ergonomic factor lever distance L for (A) L = 0, (B) L = 50 mm, and (C) L = 100 mm.

Three ergonomic factors were evaluated in this study:

(1) Lever orientation (ψ): the range of motion for supination and pronation was limited to ±30°, where supination is +30° (Figure 5A), the neutral position is 0° (Figure 5B), and pronation is −30° (Figure 5C). Although Sullivan et al [27] reported that up to a certain degree of pronation, subjective discomfort is lesser and higher effort can be achieved compared to supination and neutral hand orientation [28], objective evaluation using electromyography was required.

(2) Torso angle (λ): 3 trunk orientations in the coronal plane were studied [29,30], including 45° (Figure 6A), 30° (Figure 6B), and 0° upright (Figure 6C). The maximum low reach can be achieved at 45°, while maximum stability and minimum spinal loading can be attained at 0°.

(3) Lever distance (L): the distance from the shoulder to the lever (shoulder grip length+L) was varied using a novel seat adjustment mechanism. The distance L was calculated from the lower back of the seated participant to the backrest of the wheelchair. Three levels were tested, including L=0 mm (Figure 7A), L=50 mm (Figure 7B), and L=100 mm (Figure 7C).

#### Participants and Experimental Design

Seven healthy participants (S1–S7) were selected for the surface electromyographic study. The anthropometric data of the participants is shown in Table 3.

**Table 3.** Anthropometric characteristics of participants in the surface electromyography (sEMG) study.

Participants	Age (years)	Upper arm length (cm)	Lower arm length (cm)	BMI
S1	28	32	24	22.6
S2	33	34	23	26
S3	30	34	26	21
S4	31	36	28	20.7
S5	31	36	27	22.5
S6	33	32	24	24
S7	31	37	29	25

A 3-factor, 3-level experimental design was used. The factors and their levels are shown in Table 4. A full factorial design would require 27 runs; this was reduced to a 1/3 fractional factorial design using the Taguchi method, requiring 9 experimental runs (Table 5). Each participant performed the lever push-pull action while seated in the configuration specified by each experiment.

**Table 4.** Factors and their respective levels for identifying ergonomic seating.

Factors	Level 1	Level 2	Level 3
Torso angle (λ)	45^o^	30^o^	0^o^
Lever distance (L)	0 mm	50 mm	100 mm
Lever orientation (ψ)	–30^o^	0^o^	+30^o^

**Table 5.** 1/3 fractional factorial design of experiment (Taguchi) for 3 factors, 3 levels.

Experiment number	Torso angle level (λ)	Lever distance level (L; in mm)	Lever orientation level (ψ)
E1	45^o^	0	−30^o^
E2	45^o^	50	0^o^
E3	45^o^	100	+30^o^
E4	30^o^	0	0^o^
E5	30^o^	50	+30^o^
E6	30^o^	100	−30^o^
E7	0^o^	0	+30^o^
E8	0^o^	50	−30^o^
E9	0^o^	100	0^o^

#### Instruments and Electromyography Data Recording

**Figure 8.**
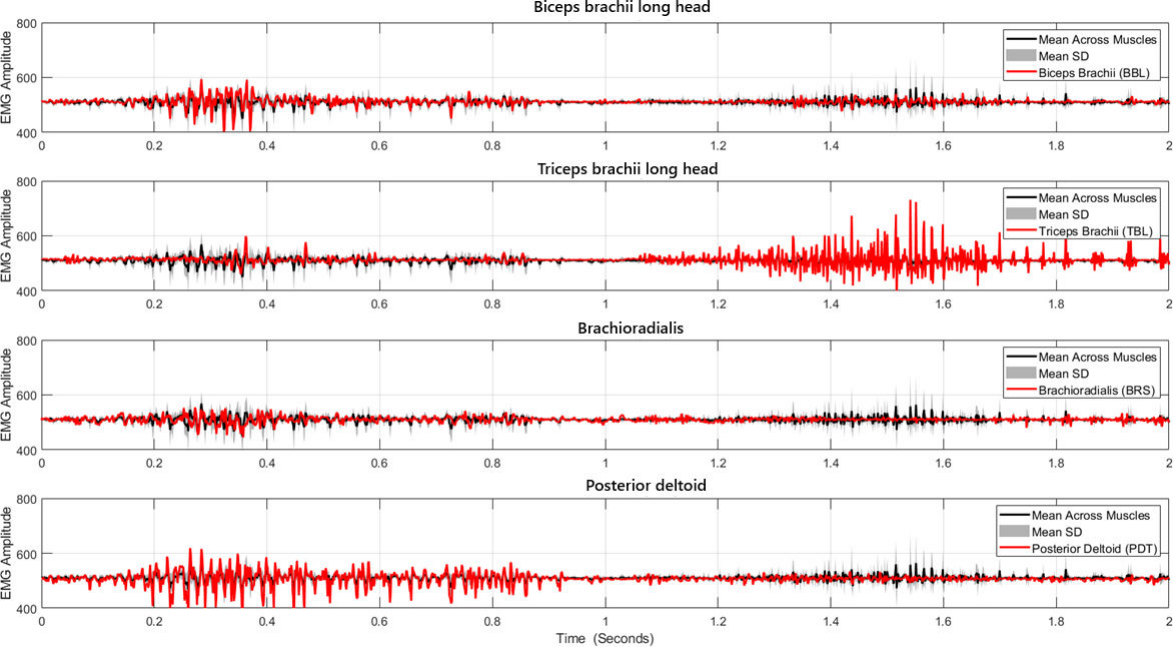
Surface electromyography (sEMG) data recorded on an ADS1299-based board for muscle groups biceps brachii long head (BBL), triceps brachii long head (TBL), brachioradialis, and posterior deltoid (PDT) for participant S1 during experiment E1.

The test wheelchair used in this study is a manually propelled stair-climbing wheelchair with a ratchet-based lever mechanism that provides a to-and-fro motion where pulling toward the chest engages the lever with the propulsion shaft, providing rotational motion to the stair-climbing wheel. For recording sEMG data, an ADS1299-based evaluation board (Texas Instruments) was used, providing 8 analog channels with a common reference, as reported in earlier sEMG studies [31-33]. These 8 channels were configured to create 4 channels with a common-mode rejection ratio for high-quality sEMG data for all 4 muscle groups as shown in Figure 8. An instrumentation gain of 11X and an overall system gain of 2420X were applied. Ultra-low impedance (<100 ohms) nickel-chromium (Ni-Cr) alloy electrodes were used with silver-silver chloride electrode (Ag-Cl) adhesive solid hydrogel at the electrode-skin interface to reduce skin impedance. The sampling frequency was 500 Hz.

**Figure 9.**
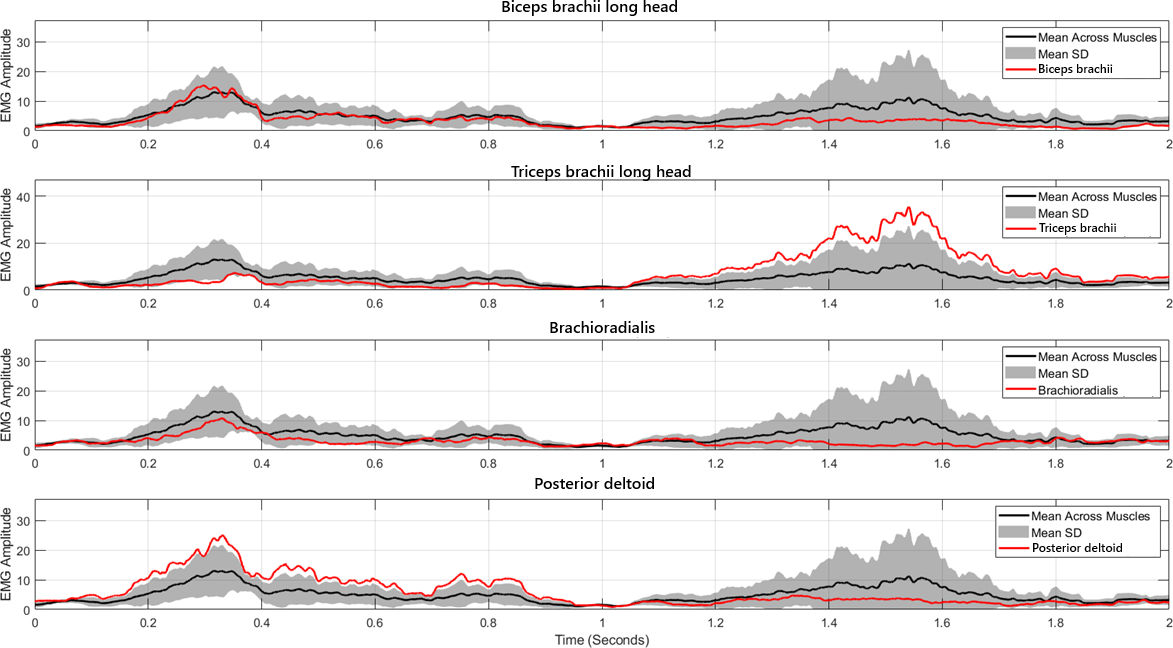
Digitally filtered data for muscle groups BBL, TBL, brachioradialis, and PDT for participant S1 during experiment E1, showing individual activation of muscles following activation timing patterns of pushing and pulling.

**Figure 10.**
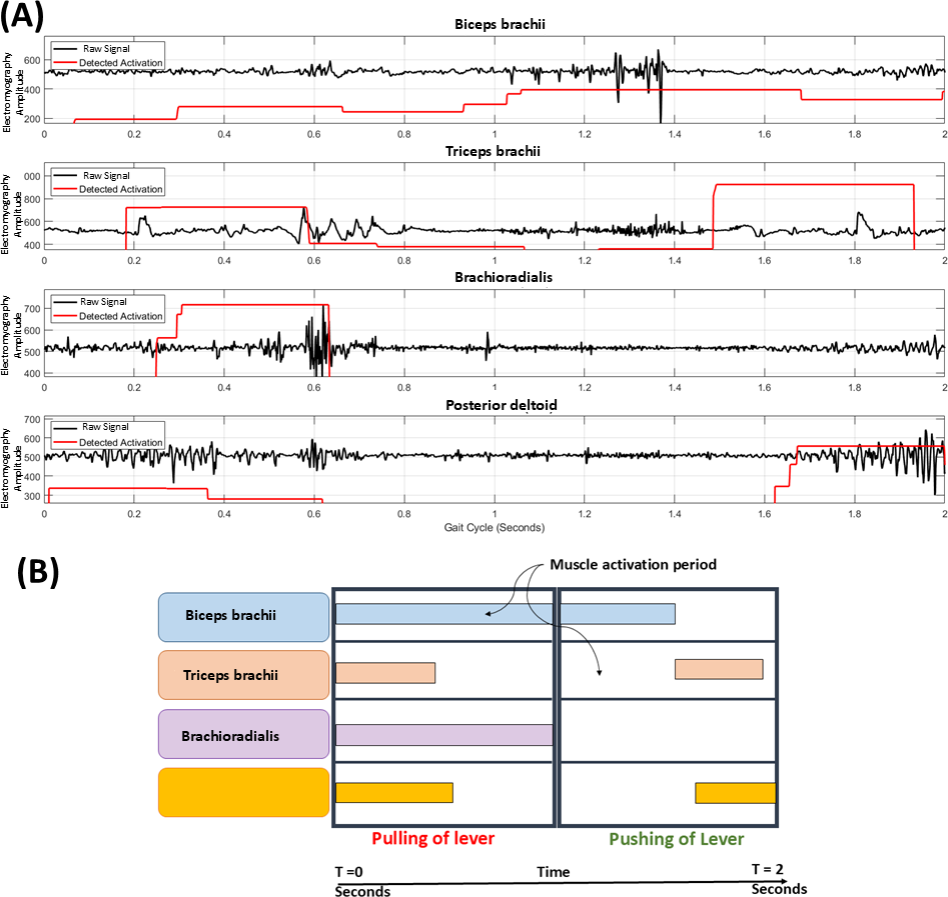
Muscle activation detection and its representation (A) Detection of muscle activation timing and amplitude threshold on a digitally processed electromyography signal (participant S1, experiment E8) using the Hilbert transform. (B) Muscle activation window in pull-push cycle of lever propulsion. BBL: biceps brachii long head; PDT: posterior deltoid; TBL: triceps brachii long head.

The raw electromyography signal was filtered using a band-pass Butterworth Infinite Impulse Response filter. The filtered electromyography signal was further processed by taking the absolute value and applying a moving average filter (window of 50 samples) as shown in Figure 9. For identification of muscle activation timing based on adaptive threshold, the Hilbert transform was used with a threshold duration of 25 samples to avoid influence of noise or other involuntary artifacts visible in Figure 10A. From the activation timing study, an activation window for the pull-push cycle is illustrated in Figure 10B.

#### Electromyography Data Processing: Maximum Voluntary Contraction

Maximum voluntary contraction (MVC) was calculated from the digitally filtered electromyography envelope with absolute values as a quantifiable parameter for normalizing the electromyography signal. The weighted average of envelope amplitude and envelope root-mean square (RMS) was used to calculate MVC [34], expressed as:

*MVC=w1 ✕ A+w2 ✕ RMS *       (6)

Where,A is the maximum amplitude of the electromyography envelope given by

*A=max (X) –min (X) *        (7)

 $X=\{x_{1},x_{2},x_{3}\ldots \ldots x_{n}\}$; X is the dataset of filtered electromyography signal recorded over time t.

*x_i_* represents the amplitude of the electromyography signal at i-th time point.

RMS means root mean square of dataset X.

Following Merletti and Parker (2004) [34], the weights were set as w1=0.7 and w2=0.3, prioritizing sensitivity to maximum activation for detecting full recruitment peaks while complementing it with RMS stability to mitigate outlier effects.

Muscle coordination patterns were assessed using the Pearson correlation coefficient “r” to measure the linear relationship between MVC values of muscle pairs [35]. The coefficient is given by:


$$r=\frac{\sum_{i=1}^{n}\left(x_{i}-\overset{-}{x}\right)\left(y_{i}-\overset{-}{y}\right)}{\sqrt{\sum_{i=1}^{n}{\left(x_{i}-\overset{-}{x}\right)}^{2}\sum_{i=1}^{n}{\left(y_{i}-\overset{-}{y}\right)}^{2}}}$$

where x_i_ and y_i_ are the MVC values for 2 muscles (eg, anterior deltoid and biceps brachii) across all observations, and $\overset{-}{x}$ and $\overset{-}{y}$ are their means.

#### Main Effect and Interaction Effect Analysis

Quadratic regression models were fitted for each ergonomic factor–muscle response relationship to characterize the design space. Main effects and interaction effects (AB: λ×L; AC: λ×ψ; BC: L×ψ) were computed by averaging MVC values across paired factor combinations. Interaction effects were visualized using line plots and heatmaps. 3D surface plots were generated to delineate the ergonomic design space. Statistical significance of correlations between anthropometric dimensions and task performance was assessed at *P*<.05.

### Ethical Considerations

This study was approved by the Institutional Ethics Committee (IEC) of the Indian Institute of Technology Kanpur (IEC Communication Number: IITK/IEC/2024-25/I/29, Date of Approval: June 20, 2024). The study followed the protocol titled “Design and Development of a multi-functional assistive device for restricted Mobility and Rehabilitation.” All procedures involving human participants adhered to the institutional ethical standards and the 1964 Helsinki Declaration and its later amendments or comparable standards. Written informed consent was obtained from all participants, who were informed of objectives, procedures, risks, and their right to withdraw without consequences. No identifying information is included here; data were anonymized and securely stored per institutional and national privacy guidelines. Participants received no financial compensation. For those with paraplegia, extra precautions ensured comfort and safety during testing.

## Results

### Part A: Plane Surface Movement Results

#### Task Completion Times

The time taken for each of the 9 participants to complete each task on the simulated course is presented in Table 6.

**Table 6.** Time taken for each participant to complete the task (in seconds).

Task	Participants								
	S1	S2	S3	S4	S5	S6	S7	S8	S9
C^a^	5	4	3.5	3	3.5	4.2	4	3.5	5.3
R^b^	12.7	14.5	15	12.5	14	18	13.8	16.4	16.2
T1^c^	21.4	23	25.4	19.5	20.5	24	20	19	22
F1^d^	6	7.2	8.1	6.3	5	4.5	4.3	4	5.5
R1^e^	1.9	2.5	2	2	2	2	2	2.3	2.8
N^f^	10.4	9.8	6	8	7	9.5	6.5	8.3	9.9
F2^g^	2	2	2	2.8	2	2.5	2	2.2	2.3
R2^h^	5.5	3	3.4	4	4	4	5	3.2	4.4
T2^i^	9.2	7	6	7.3	8.2	9	8.5	7	7

a C: concrete.

b R: ramp.

c T1: 90° turn

d F1: granite floor.

e R1: rubber hollow carpet.

f N: narrow path.

g F2: ceramic tile floor.

h R2: rubber carpet.

i T2: gradual turn.

The ramp (R) and sharp 90° turn (T1) emerged as the most time-intensive tasks. The ramp required an average of approximately 14.8 seconds, while the 90° turn required approximately 21.6 seconds. Tasks on smooth floor surfaces (F1 and F2) and rubber carpets (R1 and R2) required relatively less time, averaging 2‐5 seconds each.

The time required to complete the 9 standardized wheelchair driving tasks, along with participant favorability ratings, is summarized below. All times are reported as mean (SE) in seconds.

Task C (straight corridor): Participants completed the task in 4.1 ± 0.9 s and rated it as moderately favorable. Task R (right turn): 14.8 (1.8)s, rated as moderately unfavorable. Task T1 (tight turn 1): 22.3 (2.1) s, rated as strongly unfavorable. Task F1 (forward maneuver 1): 5.7 (1.2) s, rated as moderately unfavorable. Task R1 (reverse 1): 2.4 (0.4)s, rated as moderately favorable. Task N (narrow passage): 8.5 (1.5) s, rated as strongly favorable. Task F2 (forward maneuver 2): 2.5 (0.5) s, rated as moderately favorable. Task R2 (reverse 2): 4.1 (0.8) s, rated as moderately favorable. Task T2 (tight turn 2): 7.8 (1.1) s, rated as moderately favorable.

These results indicate that tasks involving tight maneuvers (particularly T1) required substantially more time and received the least favorable ratings, whereas simpler forward and reverse tasks were completed quickly and viewed more positively. The pattern highlights the importance of adequate space and clear pathways in wheelchair-accessible environments.

#### Subjective Ratings and Overall Assessment

In the overall subjective response study for all 9 participants evaluating wheelchair attributes on a visual analog scale from 1 to 10, overall ride comfort received the lowest mean rating of 3.33 (SD 1.58), indicating areas for potential improvement in user experience during mobility. Wheelchair stability was rated moderately higher at a mean of 5.44 (SD 1.74), while driving control emerged as the strongest attribute with the highest average score of 6.00 (SD 1.22), suggesting effective handling and responsiveness in the device's design. Perception of the wheelchair scored a mean of 3.89 (SD 1.17), reflecting possibly mixed sensory feedback, whereas maneuverability—potentially encompassing response aspects—achieved a solid mean of 5.11 (SD 1.17), highlighting reliable navigation capabilities as depicted in the bar graph with error bars representing SDs. The correlation between task difficulty (higher time and low level of easiness) and anthropometric dimensions of the participants was not statistically significant (* P*=.04), indicating that task difficulty could be directly associated with wheelchair design variables rather than participant characteristics.

### Part B: sEMG and MVC Results

#### Electromyography Amplitude and MVC Values

The average electromyography amplitude and RMS of all 7 partiticipants for each muscle group, along with calculated MVC values, are presented in Table 7. There was no significant statistical correlation between the anthropometric features and electromyography activity in terms of MVC (*P*=.04). The maximum electromyography amplitude for each muscle averaged from all 9 experiments is illustrated in Figure 12A. Muscle coordination patterns derived from MVC data using the Pearson correlation coefficient are shown in Figure 12B.

**Table 7.** Average electromyography amplitude and root mean square (RMS) of all 7 participants for each muscle group along with their calculated maximum voluntary contraction (MVC) values.

Experiment	Electromyography average amplitude				Electromyography root mean square				MVC			
	BBL^a^	TBL^b^	Brachioradialis	PDT^c^	BBL	TBL	Brachioradialis	PDT	BBL	TBL	Brachioradialis	PDT
E1	69.3	94.4	149.6	132.3	7.8	12.2	22.3	17.4	50.85	69.74	111.41	97.83
E2	55.5	74.8	179.0	95.0	7.3	10.5	13.24	12.4	41.04	55.51	129.272	70.22
E3	56.8	105.2	136.1	88.3	9	11.9	14.3	11.5	42.46	77.21	99.56	65.26
E4	52.9	75.1	128.1	82.6	8.3	12.0	13.0	10.0	39.52	56.17	93.57	60.82
E5	59.0	76.5	129.8	83.9	8.6	10.3	15.0	12.0	43.88	56.64	95.36	62.33
E6	56.7	102.5	130.9	121.3	9.0	13.9	16.8	19.0	42.39	75.92	96.67	90.61
E7	47.8	72.9	85.6	77.6	8.6	11.5	11.6	10.1	36.04	54.48	63.4	57.35
E8	48.7	55.8	75.1	62.1	8.0	9.0	11.3	10.0	36.49	41.76	55.96	46.47
E9	56.6	64.0	132.4	64.0	10.8	9.5	14.8	9.7	42.86	47.65	97.12	47.71

a BBL: biceps brachii long head.

b TBL: triceps brachii long head.

c PDT: posterior deltoid.

**Figure 11.**
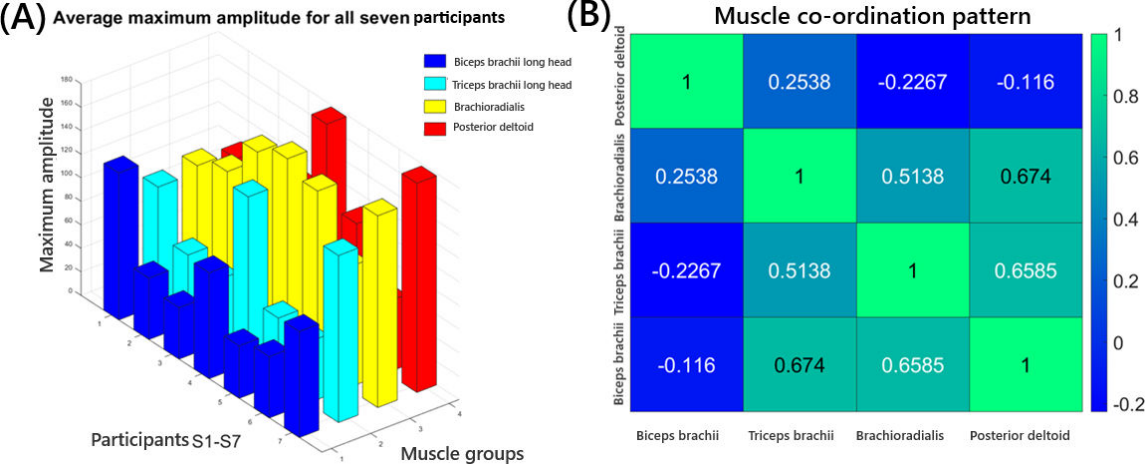
Electromyography voltage output for all 4 muscle groups of 7 participants. (A) Average maximum amplitude of electromyography signal at different muscle groups for all 7 participants (S1-S7) calculated from all 9 experiments (E1-E9). (B) Muscle coordination pattern calculated using MVC values. BBL: biceps brachii long head; PDT: posterior deltoid; TBL: triceps brachii long head.

#### Quadratic Regression Analysis

Quadratic regression models were fitted for each factor-muscle relationship. The regression equations, residual sum of squares, and coefficients of determination are presented in Table 8.

**Table 8.** Quadratic regression analysis and its parameters for muscle group response and ergonomic factors.

Relation	Equation	Residual sum of squares	Coefficient of determination (%)
BBL^a^-lever distance	BBL=42.14‐0.0710×L+.000753×L^2^	4.96	4.8
BBL-torso angle	BBL=38.46+.0658×ʎ+0.001659×ʎ^2^	3.98	38.7
BBL-lever orientation	BBL=41.14‐0.04083×ψ+0.000976×ψ^2^	4.91	6.8
TBL^b^-lever distance	TBL=60.13‐0.4210×L+.004890×L^2^	11.79	30.6
TBL-torso angle	TBL=47.96+.6270×ʎ−0.00429×ʎ^2^	9.81	52.0
TBL- lever orientation	TBL=53.11+.0051×ψ+0.01057×ψ2	13.05	15.1
Brachioradialis- lever distance	Brachioradialis=89.46+.0796×L+.000036×L^2^	25.41	2.6
Brachioradialis-torso angle	Brachioradialis=72.16+.470×ʎ+0.00992×ʎ^2^	15.35	64.5
Brachioradialis-lever orientation	Brachioradialis=106.7‐0.0318×ψ −0.02177×ψ2	23.11	19.4
PDT^c^-lever distance	PDT=72.00‐0.4517×L+.004103×L^2^	19.27	9.6
PDT-torso angle	PDT=50.51+.863×ʎ −0.00571×ʎ^2^	14.43	49.3
PDT-lever orientation	PDT=59.58‐0.2776×ψ −0.01155×ψ2	17.48	25.6

a BBL: biceps brachii long head.

b TBL: triceps brachii long head.

c PDT: posterior deltoid.

#### Main Effects on MVC

**Figure 12.**
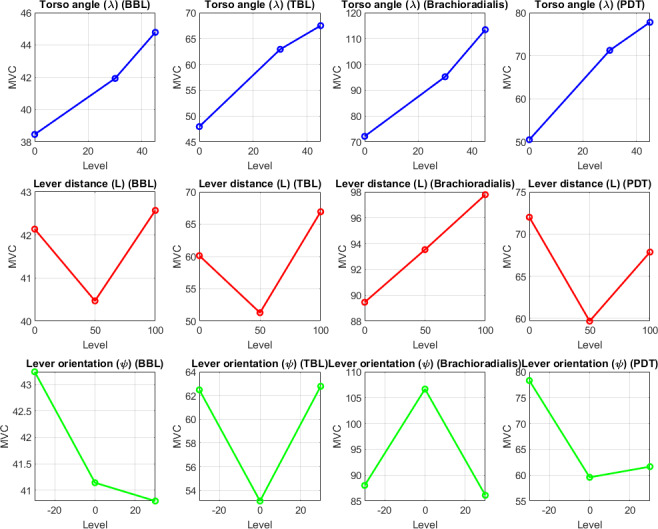
Main effect plot derived from the mean of maximum voluntary contraction (MVC) values with respect to ergonomic factors for MVC of biceps brachii long head (BBL), triceps brachii long head (TBL), brachioradialis, and posterior deltoid (PDT) changes with change in torso angle (λ), lever distance (L), and lever orientation angle (ψ). BBL: biceps brachii long head; MVC: maximum voluntary contraction; PDT: posterior deltoid; TBL: triceps brachii long head.

The main effects of the 3 ergonomic factors on MVC for each muscle group are summarized in Table 9 and visualized in the main effect plots (Figure 12).

**Table 9.** Main effects on maximum voluntary contraction values for factor A, B, and C.

Muscle	Level 0° (straight)	Level 30° (inclined)	Level 45° (highly inclined)
Factor A: torso angle (λ)			
BBL^a^	38.46	41.93	44.78
TBL^b^	47.96	62.91	67.49
Brachioradialis	72.16	95.20	113.41
PDT^c^	50.51	71.25	77.77
Muscle	Level 0 mm (farthest)	Level 50 mm (neutral)	Level 100 mm (closest)
Factor B: lever distance (L)			
BBL	42.14	40.47	42.57
TBL	60.13	51.30	66.93
Brachioradialis	89.46	93.53	97.78
PDT	72.00	59.67	67.86
Muscle	Level –30° (pronation)	Level 0° (neutral)	Level 30° (supination)
Factor C: lever orientation (ψ)			
BBL	43.24	41.14	40.79
TBL	62.47	53.11	62.78
Brachioradialis	88.01	106.65	86.11
PDT	78.30	59.58	61.65

a BBL: biceps brachii long head.

b TBL: triceps brachii long head.

c PDT: posterior deltoid.

For torso angle (λ), MVC increased with inclination across all muscles, including BBL from 38.46 to 44.78, TBL from 47.96 to 67.49, brachioradialis from 72.16 to 113.41, and PDT from 50.51 to 77.77, with a straight torso (0°) yielding the lowest strain across all muscles, particularly brachioradialis. For lever distance (L), BBL remained relatively stable (40.47‐42.57, lowest at 50 mm), TBL was optimized at 51.30 (50 mm), brachioradialis rose from 89.46 to 97.78 with decreasing L, and PDT was lowest at 59.67 (50 mm). For lever orientation (ψ), BBL slightly favored 30° (40.79); TBL and PDT were minimized at 0° (53.11 and 59.58, respectively); and brachioradialis peaked at 0° (106.65) but dropped to 86.11 at 30°.

#### Interaction Effects

**Figure 13.**
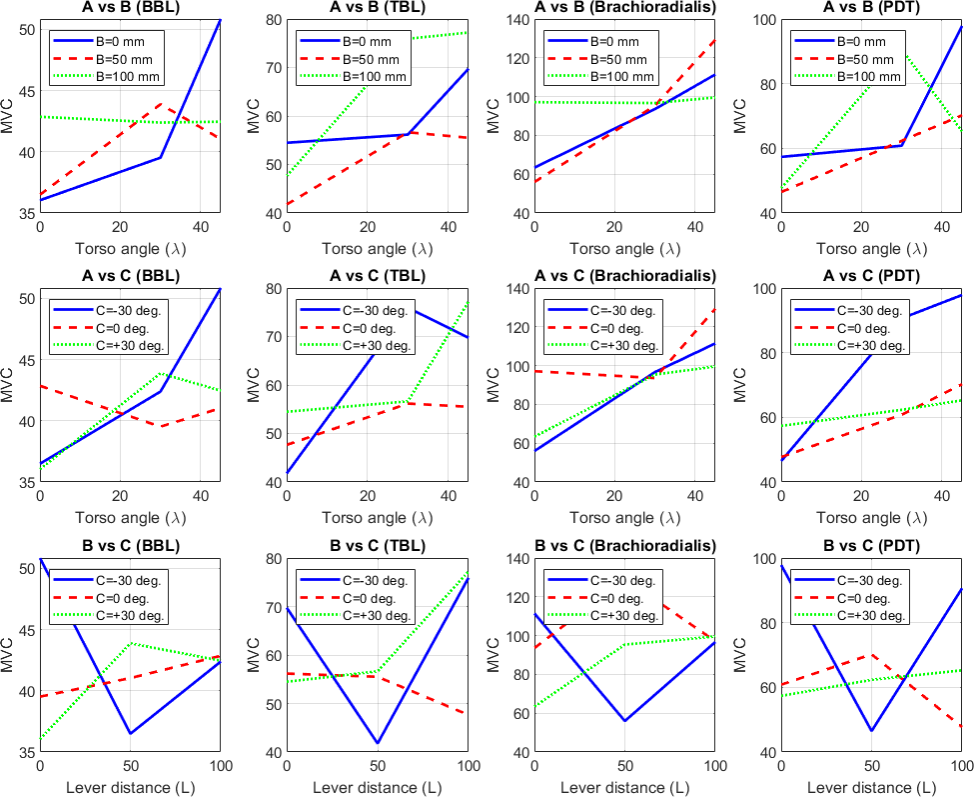
Interaction plot between ergonomics factors lever distance, torso angle, and lever orientation for biceps brachii long head (BBL), triceps brachii long head (TBL), brachioradialis, and posterior deltoid (PDT). BBL: biceps brachii long head; MVC: maximum voluntary contraction; PDT: posterior deltoid; TBL: triceps brachii long head.

**Figure 14.**
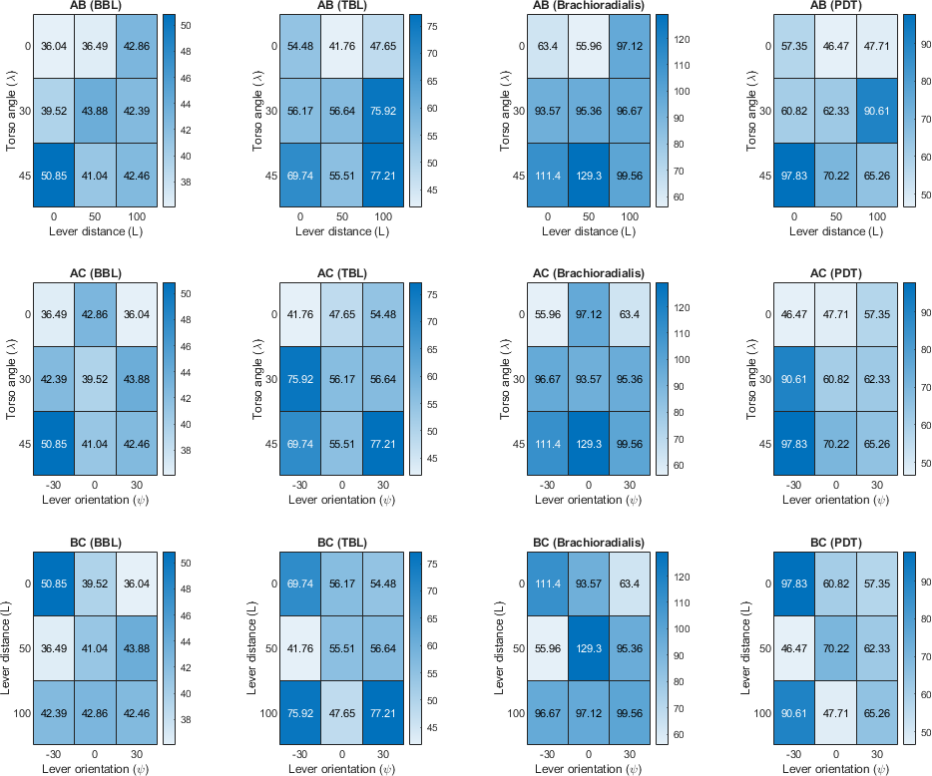
Interaction heat map between ergonomics factors lever distance, torso angle, and lever orientation for biceps brachii long head (BBL), triceps brachii long head (TBL), brachioradialis, and posterior deltoid (PDT). BBL: biceps brachii long head; MVC: maximum voluntary contraction; PDT: posterior deltoid; TBL: triceps brachii long head.

Interaction effects (AB: λ×L; AC: λ×ψ; BC: L×ψ) were computed by averaging MVC values across paired factor combinations. These interactions are visualized in line plots (Figure 13) and heatmaps (Figure 14).

For the AB interaction (Torso angle ×Lever distance), forward lean (λ=45°) combined with long lever distance (L>50 mm) produced elevated effort in BBL and TBL, with lines crossing in Figure 13 and heatmaps showing high-strain zones. Upright posture (λ=0°) at L≈50 mm maintained low effort levels.

For the AC interaction (Torso angle ×Lever orientation), supination (ψ =+30°) combined with λ=0° reduced effort for brachioradialis and PDT, while pronation (ψ = –30°) at λ=45° increased effort, shown by crossing lines and heatmap contrasts. TBL was less affected by this interaction.

For the BC interaction (Lever distance ×Lever orientation), L ≈ 50 mm with ψ =+30° minimized brachioradialis and PDT strain, while a long lever distance with ψ = –30° maximized strain. TBL effort increased with pronation (see Figure 14).

#### Design Space Optimization

**Figure 15.**
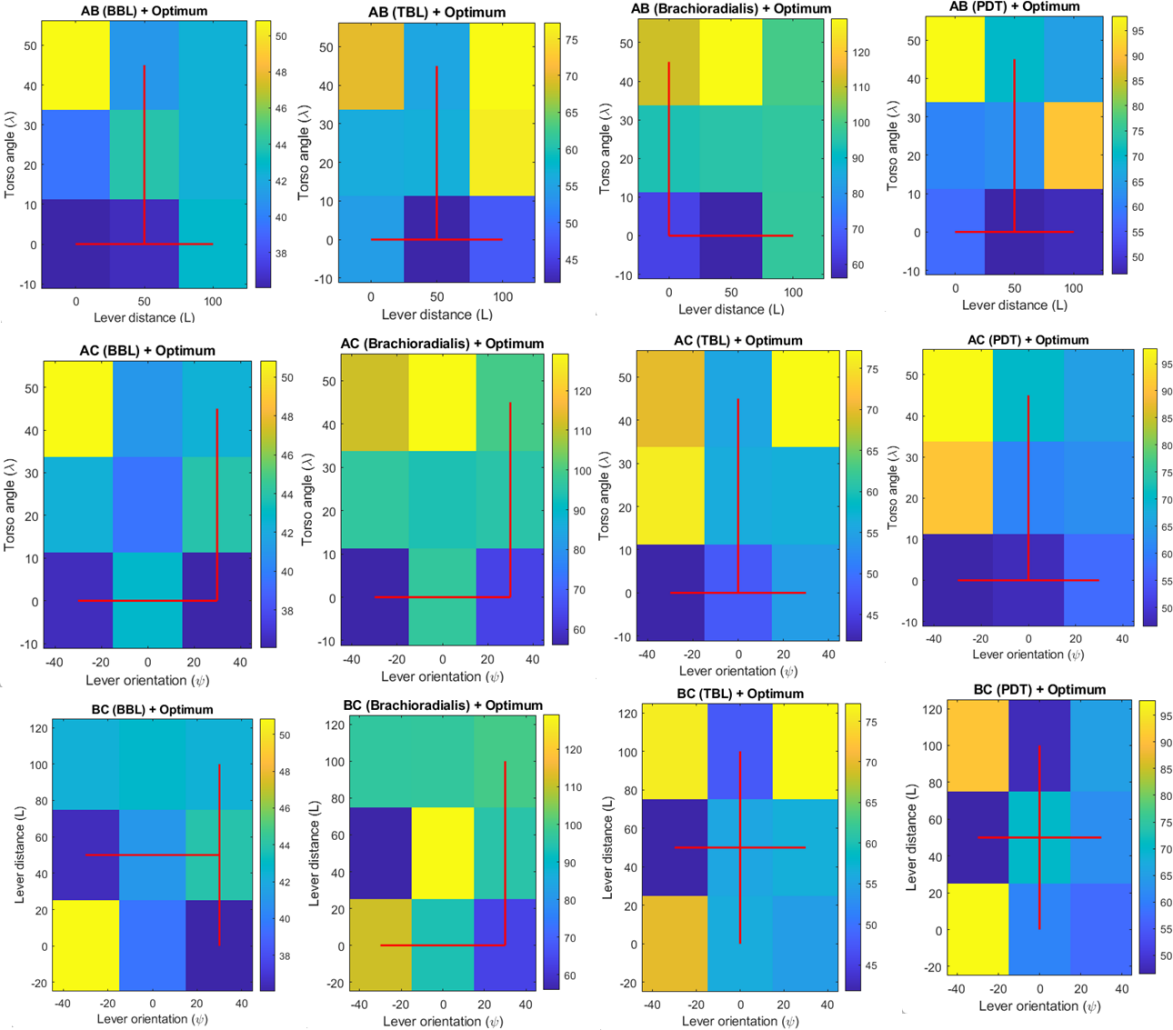
Design space heatmaps for ergonomic factors torso angle (λ) or A, lever distance (L) or B, and lever orientation (Ψ) interacting in AB, AC, and BC configurations. BBL: biceps brachii long head; MVC: maximum voluntary contraction; PDT: posterior deltoid; TBL: triceps brachii long head.

**Figure 16.**
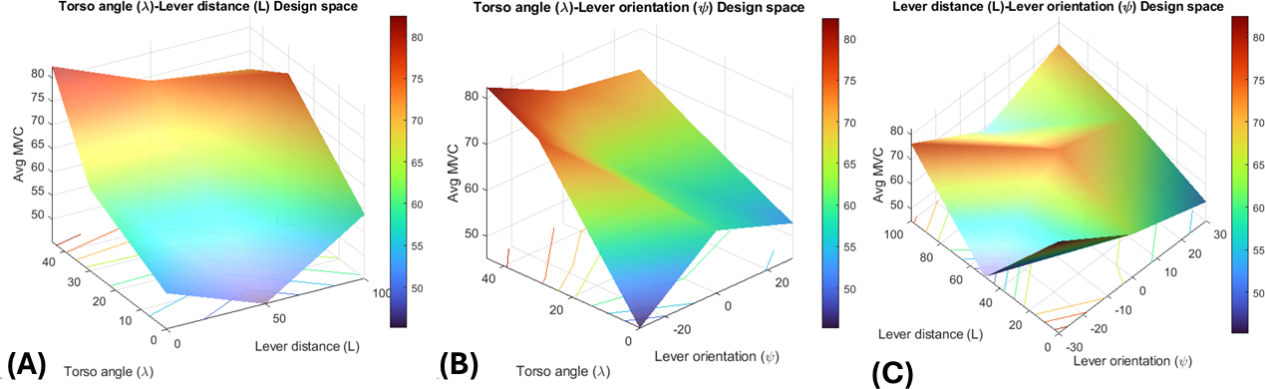
Design space for different ergonomic factors (A) torso angle (λ) and lever distance (L), (B) torso angle (λ) and lever orientation (Ψ), and (C) lever distance (L) and lever orientation (Ψ). BBL: biceps brachii long head; MVC: maximum voluntary contraction; PDT: posterior deltoid; TBL: triceps brachii long head.

The design space heatmaps (Figure 15) and their associated 3D surface plots (Figure 16) delineated the ergonomic design space. For the AB interaction (λ×L), heatmaps revealed BBL (MVC 38‐50) and PDT (50-95) with high strain at λ=45°, L=100 mm, and low strain at λ=0°, L=0 mm. The 3D surface plot peaked at 80 MVC where λ=45° and L=100 mm and dipped to 50 MVC at λ=0° and L=0 mm as shown in Figure 16A.

For the AC interaction (λ × ψ), BBL minimized strain at λ=0°, ψ=30° (supination), and PDT at λ=0°, ψ=0°, with high strain at λ=45° and ψ=−30°. The 3D plot rose to 75 MVC at λ=45°, ψ=−30° and fell to 50 MVC across λ=0° and ψ=0°–30° as shown in Figure 16B. For the BC interaction (L × ψ), BBL was optimized at L=50 mm, ψ=30°, and PDT at L=50 mm, ψ=0°. The 3D plot peaked at 80 MVC for L=100 mm, ψ=−30° and reached approximately 50 MVC near L=50 mm, ψ=0°–30°, as shown in Figure 16C.

Collectively, strain exceeded 75 MVC at extreme configurations (eg, λ=45°, L=100 mm, ψ=-30°) and dropped to approximately 50 MVC at optimal settings (eg, λ=0°, L=0‐50 mm, ψ=0°-30°).

#### Optimal Design Parameters

Optimal settings for minimizing individual muscle activity are shown in Table 10. These preferences vary by muscle but consistently favor a straight torso.

**Table 10.** Optimum levels for minimum muscle activity for all 4 muscle groups.

Muscle	Torso angle (λ)	Lever distance (L)	Lever orientation (ψ)
BBL^a^	0° (Straight)	50 mm (Neutral)	30° (Supination)
TBL^b^	0° (Straight)	50 mm (Neutral)	0° (Neutral)
Brachioradialis	0° (Straight)	0 mm (Farthest)	30° (Supination)
PDT^c^	0° (Straight)	50 mm (Neutral)	0° (Neutral)

a BBL: biceps brachii long head.

b TBL: triceps brachii long head.

c PDT: posterior deltoid.

Averaging across all muscles for a generalized design, the overall ergonomic recommendation is presented in Table 11 along with optimal wheelchair design parameters in Table 12.

**Table 11.** Overall ergonomic recommendation (averaged across muscles).

Parameter	Optimal setting
Torso angle (λ)	0.0° (Straight)
Lever distance (L)	37.5 mm (Between farthest and neutral)
Lever orientation (ψ)	15.0° (Between neutral and supination)

**Table 12.** Optimal wheelchair design parameters.

Muscle	Torso angle (in degrees)	Lever distance (mm)	Lever orientation (in degrees)	Torso angle	Lever distance	Lever orientation
BBL^a^	0	50	30	Straight	Neutral	Supination
TBL^b^	0	50	0	Straight	Neutral	Neutral
Brachioradialis	0	0	30	Straight	Farthest from lever	Supination
PDT^c^	0	50	0	Straight	Neutral	Neutral
Overall	0	37.5	15	Straight	Between farthest and neutral	Between Neutral and Supination

a BBL: biceps brachii long head.

b TBL: triceps brachii long head.

c PDT: posterior deltoid.

## Discussion

### Summary of Key Findings

This study systematically evaluated the ergonomic factors influencing muscle activity and user comfort in a stair-climbing wheelchair design through a dual-methodology approach. In Part A, the plane surface movement study revealed that the ramp and sharp 90° turn were the most challenging tasks, directly attributable to the wheelchair’s weight and large dimensions. In Part B, the sEMG analysis demonstrated that a straight torso (λ=0°) consistently minimized muscle strain across all four muscle groups, with an overall ergonomic recommendation of λ=0°, L=37.5 mm, and ψ=15° to balance strain reduction across all muscles.

### Design Implications of the Plane Surface Movement Study

The lack of statistical correlation between task difficulty and anthropometric dimensions (* P*=.04) confirms that any difficulty in performing a task can be directly associated with wheelchair design variables, which is the primary goal of this ergonomic assessment. The increase in time and effort at the ramp (R) can be directly correlated with the wheelchair weight increase due to the stair-climbing apparatus installed along with the plane-surface wheelchair. A significant amount of deadweight (net weight of 65 kg) is carried along when the plane surface mode is active. The next iteration in design will require a detailed study of strength of materials to remove as much weight as possible without compromising strength and durability.

Similarly, the sharp 90° turn (T1) required extreme maneuverability skills at the user’s side. The calculated turning distance for a 90° turn of approximately 1450 mm is a direct consequence of the overall length and width of the wheelchair, which plays a critical role in maneuverability and steering experience. The form factor must be reduced not only for addressing the sharp turn problem but also for movement through narrow spaces (Task N), which also took significant time although it had a contradictorily favorable subjective response.

The low comfort scores, particularly during plane surface movement, were attributed to the slightly tilted seat design that, while helping maintain the wheelchair user’s center of gravity away from the descent direction during stair-climbing, causes considerable discomfort during plane surface movement. These findings highlight the inherent design tension between stair-climbing functionality and plane surface comfort, suggesting that future designs should incorporate a more dynamic seat tilt adjustment mechanism. Importantly, any design changes inferred from the plane surface movement study must not compromise the primary stair-climbing function.

### Interpretation of sEMG Findings and Rationale for Optimal Parameters

The optimal design parameters (λ=0°, L=37.5 mm, and ψ=15°) were systematically determined through Taguchi design of experiments analysis, representing a carefully considered balance of biomechanical efficiency, user comfort, and practical manufacturability.

Torso angle (λ=0°): the neutral torso position minimizes shoulder abduction and maintains a natural arm movement trajectory, reducing rotator cuff stress. The straight torso position showed significant reduction in brachioradialis activation compared to forward-leaning postures (λ=15°, 30°), indicating lower muscular effort. This finding aligns with previous research on wheelchair propulsion biomechanics showing that neutral spine positioning reduces cumulative shoulder joint loading and decreases the risk of overuse injuries. As torso angle increases, muscle effort rises across the forearm (brachioradialis), arm (BBL and TBL), and shoulder (PDT) groups due to anterior torso displacement. The prototype’s backrest design mitigates fatigue, with the 0° posture proving optimal during stair-climbing compared to forward-leaning positions.

Lever distance (L=37.5 mm): this distance provides optimal mechanical advantage while maintaining comfortable reach, derived from anthropometric data analysis and biomechanical modeling. Shorter distances resulted in cramped postures and increased BBL activation, while longer distances showed increased PDT activation, suggesting compensation through shoulder muscles. The 37.5 mm distance represents the optimal compromise minimizing total muscular activation across all monitored muscle groups. Prior literature suggests that extreme upper limb reaches elevate MSD risk, a hypothesis confirmed by these results: muscle effort increased with greater L for most muscles.

Lever orientation (ψ = 15°): this slight supination optimizes weight distribution, provides postural support, and maintains shoulder joint alignment. The brachioradialis, BBL, and PDT exhibited the lowest effort at +30° (supination), while TBL effort peaked during the pronation-to-supination transition due to flexion demands. An outward lever orientation consistently reduces muscle activity, lowering fatigue and MSD risk. The 15° angle balances stair-climbing stability requirements with plane surface comfort; neutral seating (0°) showed marginally lower muscle activation but compromised stability during stair-climbing, while greater supination angles (30°) increased discomfort during prolonged sitting and reduced forward reach capability.

The interaction effects, visualized through heatmaps and 3D surface plots, underscored the interdependence of these factors. The interaction plots helped identify critical regions where the combined effects of ergonomic factors diverge, aiding in making informed decisions about the physical dimensions of the wheelchair. For instance, the AB interaction plot showed that forward lean (λ=45°) combined with long lever distance (>50 mm) should be avoided as it spikes effort, while upright posture at L≈50 mm keeps effort low. The consistency between heatmaps and 3D surfaces highlights an ergonomic sweet spot that reduces muscle effort and fatigue, offering clear guidance for refining wheelchair design to enhance user comfort and efficiency during stair-climbing tasks.

The modular design architecture permits adjustment of all 3 parameters to accommodate users with different anthropometric characteristics, strength capabilities, and functional requirements. Individual optimization may be necessary for users at anthropometric extremes or with specific upper extremity limitations.

### Weight Reduction Feasibility and Future Design Strategies

This prototype weight of 65 kg represents a significant barrier to usability, particularly for flat surface maneuverability and transportation. This weight is substantially higher than conventional manual wheelchairs (15‐25 kg) and constitutes a primary design limitation requiring immediate attention. Weight reduction strategies under investigation include the following:

1. Material substitution: replacing steel frame components with aerospace-grade aluminum alloys (estimated 8‐12 kg reduction) and carbon fiber composites for non–load-bearing elements (estimated 3‐5 kg reduction).
2. Mechanism optimization: redesigning the transformation mechanism to eliminate redundant components and implementing topology optimization for load-bearing structures (estimated 4‐6 kg reduction).
3. Component integration: combining multiple discrete parts into unified assemblies to reduce fastener count and material overlap (estimated 2‐3 kg reduction).

Preliminary engineering analysis suggests a realistic target weight of 45‐50 kg is achievable through these combined strategies, representing a 23%‐31% reduction. This improved weight would significantly enhance maneuverability while maintaining structural integrity for stair-climbing operations. Cost-benefit analysis and finite element modeling are ongoing to validate these approaches prior to next-generation prototype development.

### Safety Systems, Descending Mechanism, and Wheelchair Adjustability

While this manuscript focuses on ergonomic assessment during upward stair-climbing and plane surface movement, the wheelchair incorporates comprehensive safety and functional systems essential for complete operational capability:

1. Descending mechanism: stair descent uses a controlled ratchet-and-pawl braking system integrated with the stair-climbing wheel assembly. The mechanism permits incremental wheel rotation (step-by-step descent) while preventing uncontrolled backward movement. User-activated brake levers provide variable descent speed control (range: 1‐4 steps per minute). Antitip sensors provide auditory feedback when the center of gravity approaches the instability threshold.
2. Braking systems: dual independent braking systems ensure redundancy and safety. Primary system lever-operated disc brakes on plane-surface wheels providing immediate stopping capability. Secondary system electromagnetic parking brakes preventing rollback during stationary periods. Emergency brake activation requires <1.5-second response time from full speed, meeting safety standards for powered mobility devices.
3. Adjustability features: the modular design permits extensive customization, including seat height adjustment (400‐550 mm from ground, 50 mm increments), backrest angle adjustment (0‐30° recline, 5° increments), footrest height and angle adjustment, lever position adjustment (radial distance 300‐500 mm, angular position ±30°), and seat width adjustment (400‐500 mm) to accommodate different pelvic widths. All adjustments use tool-free quick-release mechanisms for rapid configuration changes.

Comprehensive documentation of these systems, including mechanical specifications, safety testing protocols, and user operating procedures, will be presented in subsequent publications focused specifically on mechanical design and safety validation.

### Strengths and Limitations

This study has several strengths. It represents one of the first pilot studies to integrate surface electromyography with subjective usability assessment for a stair-climbing wheelchair, providing both user-perceived comfort data and objective biomechanical evidence. The Taguchi-based experimental design efficiently identified optimal ergonomic parameters while minimizing the number of experimental runs. The inclusion of participants with paraplegia, though limited in number, provided critical real-world user validation.

However, several limitations must be acknowledged. The relatively small sample size (n=9 for plane surface movement, including 2 participants with paraplegia; n=7 for sEMG study) is appropriate for this pilot investigation but limits generalizability. Future studies should include larger, more diverse participant groups, including various disability types, severity levels, and demographic characteristics, to establish comprehensive design guidelines.

A significant limitation is the inclusion of only male participants in the anthropometric survey. Female and gender-diverse individuals have different anthropometric characteristics, muscle strength distributions, and ergonomic requirements [36]. Studies have shown that female wheelchair users often experience different pressure distribution patterns, grip strength capabilities, and shoulder biomechanics during propulsion [37]. Future iterations should conduct separate anthropometric studies for different gender groups to ensure optimal comfort, safety, and usability for all users. Additionally, consideration must be given to pregnancy-related ergonomic requirements, hormone-related strength variations, and gender-specific injury risk factors that may influence long-term wheelchair use patterns.

The controlled experimental environment, while necessary for this initial validation study, limits ecological validity. Real-world wheelchair use involves additional challenges including uneven terrain, weather conditions, unpredictable obstacles, crowded spaces, and cognitive demands from environmental distractions [38]. Future research should include (1) field testing in actual urban environments under naturalistic conditions; (2) long-term usability studies capturing daily use patterns and cumulative physical demands; (3) assessment under various weather and lighting conditions to evaluate all-season functionality; and (4) evaluation of cognitive load during real-world navigation tasks, including wayfinding, obstacle avoidance, and multitasking demands typical of community mobility. Long-term usability, durability, and cumulative physical effects require extended longitudinal studies beyond the scope of this pilot investigation, such as the impact of materials for backrest, seat, and lever handle [39-41].

### Conclusion

This research provides critical design feedback for the stair-climbing wheelchair. Following a generic product design and development philosophy, the goal was to identify design variables influenced by usability testing. The most basic ergonomic assessment of a wheelchair that is, its reach and clearance, has been established through a detailed anthropometric survey of 20 participants with 5th and 95th percentile data guiding design dimensions.

The plane surface movement study demonstrated that the ramp and sharp 90° turn were the most challenging tasks, attributed to the wheelchair’s 65 kg weight and large dimensions requiring approximately 1450 mm for a 90° turn. Subjective feedback indicated low satisfaction, particularly for comfort, due to the tilted seat design. These findings highlight the need for design improvements, including weight reduction, a more compact form factor, and seat tilt adjustments, all while preserving stair-climbing functionality.

The lever-propulsion sEMG study systematically evaluated three ergonomic factors. Key findings indicate that a straight torso (0°) consistently minimizes strain, particularly for brachioradialis. A neutral lever distance (approximately 50 mm) reduced effort for most muscles, while neutral to slightly supinated orientations (0°-30°) generally optimized performance. Interaction effects guided an overall ergonomic recommendation of λ=0°, L=37.5 mm, and ψ=15°. These settings offer a balanced, practical solution for minimizing fatigue and enhancing user comfort. The modular wheelchair design permits customization for diverse user populations, and future research should include larger, gender-diverse participant groups and real-world validation studies, strengthening the foundation for ergonomic wheelchair optimization.

## Abbreviations

BBL: biceps brachii long head
CPWD: Central Public Works Department
DHM: digital human model
MSD: musculoskeletal disorder
MVC: maximum voluntary contraction
PDT: posterior deltoid
RMS: root-mean square
sEMG: surface electromyography
TBL: triceps brachii long head
